# Ecology and multilevel selection explain aggression in spider colonies

**DOI:** 10.1111/ele.12622

**Published:** 2016-06-06

**Authors:** Jay M. Biernaskie, Kevin R. Foster

**Affiliations:** ^1^ Department of Plant SciencesUniversity of OxfordSouth Parks RoadOxfordUK OX1 3RBUK; ^2^ Department of ZoologyUniversity of OxfordSouth Parks RoadOxfordUK OX1 3PSUK

**Keywords:** Adaptation, AIC, *Anelosimus studiosus*, animal personality, competition, group selection, inclusive fitness, information theoretic, kin selection, model‐based inference

## Abstract

Progress in sociobiology continues to be hindered by abstract debates over methodology and the relative importance of within‐group vs. between‐group selection. We need concrete biological examples to ground discussions in empirical data. Recent work argued that the levels of aggression in social spider colonies are explained by group‐level adaptation. Here, we examine this conclusion using models that incorporate ecological detail while remaining consistent with kin‐ and multilevel selection frameworks. We show that although levels of aggression are driven, in part, by between‐group selection, incorporating universal within‐group competition provides a striking fit to the data that is inconsistent with pure group‐level adaptation. Instead, our analyses suggest that aggression is favoured primarily as a selfish strategy to compete for resources, despite causing lower group foraging efficiency or higher risk of group extinction. We argue that sociobiology will benefit from a pluralistic approach and stronger links between ecologically informed models and data.

## Introduction

A major goal of sociobiology is to understand the extent to which adaptations are good for the individual and/or good for the group. Most evolutionary biologists agree that between‐group selection (acting at the group level) can occur to promote cooperative traits with group‐level benefits (Lehmann *et al*. 2007; Lion *et al*. 2011; Marshall 2011; Frank 2013; Gardner 2015a). However, there is always the question of whether within‐group selection (acting at the individual level) will inhibit these traits and destroy group‐level adaptation (Williams 1966; Dawkins 1976; Gardner & Grafen 2009). Abstract debates over methodology and the relative importance of within‐ vs. between‐group selection have led to much confusion in this area (Foster 2009; Okasha 2010; Birch & Okasha 2015). We believe there is an urgent need to refocus these discussions on real biological systems, where alternative models can be evaluated with empirical evidence.

In this spirit, an interesting test case has emerged in a high‐profile study of the group‐living spider *Anelosimus studiosus*, in which colony‐level aggression has been interpreted as a group adaptation (Pruitt & Goodnight 2014a). Females of *A. studiosus* can be classified as either aggressive or docile, and the composition of naturally occurring colonies varies in a site‐specific manner. Pruitt & Goodnight (2014a) found that in low‐resource sites, the proportion of aggressive females within a colony declines with colony size (number of females in the colony), whereas in high‐resource sites, it increases with colony size. Moreover, they found that experimental populations with various colony sizes and compositions changed to resemble the site‐specific patterns of naturally occurring colonies, and this was owing, in part, to the differential survival and extinction of groups. These results led to the conclusion that naturally occurring colonies exhibit the optimal aggressiveness for promoting group survival.

There are two potential problems with the group adaptation hypothesis for *A. studiosus* aggression. First, it does not explain why colony‐level aggression should vary with colony size in a site‐specific manner. For example, why should small colonies be aggressive in low‐resource sites but not in high‐resource sites? Second, as argued by recent critics, Pruitt & Goodnight’s (2014a) conclusion ignores the potential significance of within‐group selection for individual aggression (Gardner 2015b; Grinsted *et al*. 2015; Smallegange & Egas 2015). Here, we examine how incorporating within‐group selection – specifically, selection for individual competitiveness over local resources – can explain the patterns of aggression in *A. studiosus*.

We model how natural selection acting both within and between groups shapes the optimal aggressiveness of individual spiders in different colony sizes and resource environments. At the individual level, we assume that aggression helps females to compete for local resources (Pruitt *et al*. 2008; Pruitt & Riechert 2009), but this involves a cost at the group level – either reducing foraging efficiency when resources are abundant (Pruitt & Riechert 2009) or depleting resources when they are scarce, thereby risking group extinction (Pruitt & Goodnight 2014a). By incorporating this ecological detail, our models provide a striking fit to the data on aggression and colony size in *A. studiosus* populations. Our results suggest that incorporating within‐group selection for individual competitiveness is central to understanding the ecology and evolution of this system.

## Materials and Methods

### Basic model

We use a simple model of individual aggressiveness that is meant to approximate the biology of *A. studiosus*. The usual measure of female aggression in this species has a distribution that is continuous and bimodal, leading to the classification of females as either ‘aggressive’ or ‘docile’ (Pruitt & Riechert 2009). For simplicity, we ignore this apparent dimorphism and solve for a single candidate evolutionarily stable strategy (Maynard Smith & Price 1973) – that is, the level of aggression for a particular group size where, if all group members were to express this aggressiveness, selection would stop – and later, we compare this prediction with data on the average aggressiveness of females in colonies of different size. As an alternative, in the Supplementary Information, we interpret our model as predicting the optimal probability of adopting the ‘aggressive’ phenotype, and we compare this with data on within‐colony proportions of ‘aggressive’ females. Both approaches lead to the same conclusions.

We consider a very large, structured population with social interactions occurring in homogeneous groups of *n* adult female spiders. We assume that groups may be composed of family members; that social interactions are completely random within the group; and that social interaction precedes a dispersal event, in which offspring leave the group to compete in the global population. During within‐group interactions, each female expresses a genetically determined aggressiveness, which could be interpreted as a fixed level of aggression or as part of a conditional strategy, where aggressiveness is adjusted to the group size. We assume that aggressiveness affects (1) an individual's ability to compete for resources within the group and (2) the success of the group as a whole (e.g. its survival and/or number of offspring leaving the group).

We adopt a standard optimisation approach for continuously varying social traits and interactions among relatives (Taylor & Frank 1996; Frank 1998). We focus on a group in which all females express the same aggressiveness and then ask whether natural selection favours more or less aggression, or no change at all (indicating an optimum). To model the direction of selection, we consider the fitness effects of a mutant allele that changes individual aggressiveness only slightly. We assume that the mutant allele is rare in the global population; however, owing to genetic relatedness, it may be common in the local group, and so group mates may have correlated aggressiveness. We focus on a mutant individual with aggressiveness *x* and use *z* to denote the average aggressiveness of her entire group (including the focal individual herself).

Following Frank (1994), we write the focal individual's fitness as the product of two components (see also Gardner 2015b). First, we assume that her within‐group advantage in resource competition is proportional to *x/z*. Hence, if the focal individual is more aggressive than her group mates, then she can acquire more than an equal share of resources (reflecting the potential for within‐group selection). Second, the focal individual's aggressiveness contributes to a group‐level aggression trait *Z*, which we assume affects the success *G* of her group as a whole (reflecting the potential for between‐group selection). Altogether, the focal individual's fitness is

(1) w(x,z)=(x/z)G(Z),

which allows for alternative assumptions about (1) which group‐level aggression trait *Z* affects group success (we will consider the group mean *z* and the group total *zn*); and (2) the form of the relationship between the group‐level trait and group success [i.e. the shape of *G*(*Z*)].

Following Taylor & Frank (1996), the direction of selection for aggressiveness can then be can be written as

(2) $$\frac{dw}{dx}=\frac{\partial w}{\partial x}+\frac{\partial w}{\partial z}\frac{dz}{dx},$$

where all derivatives are evaluated at *x *=* z*. Viewing the focal individual as an actor, ∂*w*/∂*x* measures the benefit to herself owing to her increased competitiveness; ∂*w*/∂*z* measures the effect of her aggressiveness on the success of the whole group, including herself; and d*z*/d*x* = *R* measures the expected relatedness between the focal individual and a random individual in the whole group, including herself. This ‘whole‐group’ version of relatedness (Pepper 2000) can be viewed as a measure of the relative importance of between‐group selection compared to within‐group selection (Foster 2009; Wenseleers *et al*. 2010; Goodnight 2013). Equivalently, we can define *R *= (1/*n*) + *r*(*n *− 1)/*n*, where *r* is the expected relatedness between the focal individual and a random group mate. This ‘others‐only’ version of relatedness (Pepper 2000) can be viewed as a measure of how much the focal individual values the fitness of her group mates, as used by inclusive fitness (kin selection) theory (Hamilton 1964a,b).

We predict the optimal individual aggressiveness z* by solving for the point at which directional selection stops ${\left(dw/dx\right|}_{x=z=z^{*}}=0)$. This is the predicted aggression level that balances within‐ and between‐group selection or, equivalently, the aggression level that is optimal from an individual's inclusive fitness perspective (Gardner 2015b). For our purposes, it is particularly useful to consider model results in terms of group size *n* and others‐only relatedness *r*. Our aim is to predict how z* will vary with group size when incorporating selection for within‐group competitiveness, in comparison to the extreme case of pure group‐level adaptation. We represent group‐level adaptation by setting *r *=* *1 because this means that all group members value each other as if they were parts of the same individual (i.e. there is no within‐group conflict). In contrast, within‐group selection occurs whenever *r *<* *1 because this means that an individual values herself more than her group mates (i.e. there is within‐group conflict). The latter case represents multilevel selection because selection is occurring at both the individual and the group level.

### High‐resource models

In high‐resource sites, where prey are abundant, we assume that aggression has a negligible effect on total group resources but reduces the efficiency of the group's consumption of these resources, as individuals divert more effort towards selfish within‐group competition (consistent with evidence from Pruitt & Riechert 2009). This is an example of a ‘shared cost’ (Godfray & Parker 1992), where the costs experienced by all group members are a function of the average aggressiveness in the group (hence *Z *= *z*). Specifically, we assume that if a single individual were to increase her aggression by an amount δ, the costs experienced by all individuals (e.g. more time spent in aggressive interactions rather than foraging and brood care) become a function of *Z *+ δ/*n*. Hence, in this model, the costs of aggression are dissipated among the members of the group.

**Figure 1:**
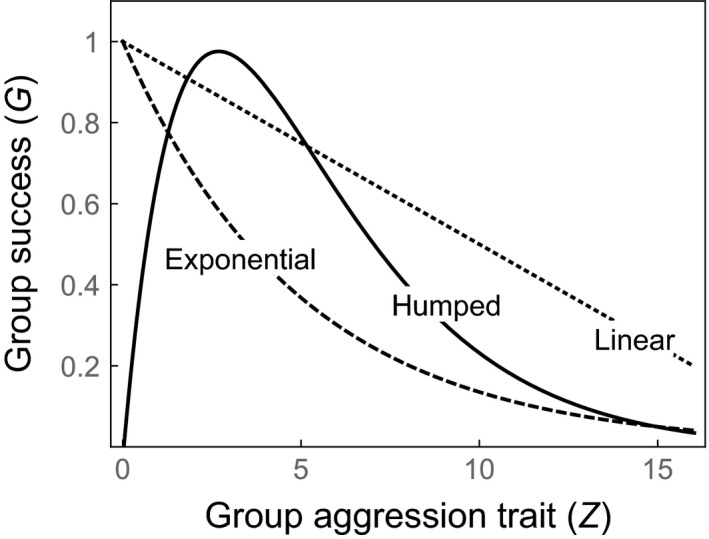
Alternative functions for group success *G*(*Z*) used in our optimality models. The ‘linear cost’ model assumes *G *=* *1 − *bZ* (shown with *b *=* *0.05); the ‘exponential cost’ model assumes *G *= exp[−*bZ*] (shown with *b *=* *0.02); and the ‘humped benefit’ model assumes *G *= *Z *× exp[−*Z*/*b*] (shown with *b *=* *2.7).

We consider three versions of the high‐resource model, based on the group success functions depicted in Fig. 1 (using *Z *= *z*): a linear decline (‘linear cost’ model; *G *=* *1 − *bz*); an exponential decline (‘exponential cost’ model; *G *= exp[−*bz*]); and a hump‐shaped function (‘humped benefit’ model; *G *= *z* exp[−*z*/*b*]). The exponential cost model allows the marginal cost of aggression to decline as the average aggressiveness *z* increases (which could occur, for example, if the frequency of aggressive interactions increases with *z* at a diminishing rate). The humped benefit model incorporates evidence that aggression can have a group beneficial effect of defending against social parasites (e.g. heterospecific spiders; Pruitt & Riechert 2011; Pruitt 2012; Wright *et al*. 2014), though we assume that this benefit will eventually be outweighed by the efficiency costs described above.

### Low‐resource models

In low‐resource sites, where prey are scarce, we assume that the main cost of aggression is to deplete the total resources (prey abundance) in the group, thereby increasing the risk of starvation and group extinction (consistent with group extinctions associated with cannibalism, as observed by Pruitt & Goodnight 2014a). This is an example of a ‘summed cost’ (Godfray & Parker 1992), where the costs experienced by all group members are a function of the total aggressiveness in the group (hence *Z *= *zn*). Specifically, we assume that if a single individual were to increase her aggressiveness by an amount δ, the costs experienced by all individuals (e.g. higher probability of extinction) would become a function of *Z *+ δ. Hence, in contrast to the high‐resource model, the costs of aggression in this model are not dissipated among the members of the group.

We consider two versions of the low‐resource model (Fig. 1, using *Z *= *zn*): ‘linear costs’ (*G *=* *1 − *b*(*zn*)) and ‘exponential costs’ (*G *= exp[−*b*(*zn*)]). We do not consider the group‐defence hypothesis here, based on evidence that social parasites are not associated with colony extinctions in low‐resource sites (Pruitt & Goodnight 2014a).

### Model fitting and assessment

We derived separate models of optimal individual aggressiveness in high‐ and low‐resource environments, based on our intuition and available evidence about these ecological settings. Accordingly, we fit each version of the high‐resource model to data on the average individual aggressiveness in high‐resource sites (216 colonies total) and each version of the low‐resource model to the corresponding data from low‐resource sites (232 colonies total) (Pruitt & Goodnight 2014a,b). We do not consider the opposite comparisons because the fit would clearly be poor (with model predictions and data going in opposite directions). When fitting all models to data, we specified *r *=* *0.25 (an estimate from Pruitt & Goodnight 2014a), corresponding to an optimal aggression level that balances within‐ and between‐group selection. For low‐resource models in particular, we added a positive constant *a* to each model, in order to account for a positive baseline level of aggression observed in low‐resource sites.

We also compared the fit of our optimality models to a simple linear regression model, *a *+ *bn*, as used by Pruitt & Goodnight (2014a). This model represents a pure group‐level adaptation hypothesis, in which the optimal group‐level aggressiveness increases or decreases linearly with group size. Although we do not consider any model of pure group‐level adaptation to be a plausible explanation for the data, we include this particular version (‘linear group adaptation’) for its significance as the current model used to explain *A. studiosus* aggression.

We fit all models by maximum likelihood and used an information‐theoretic approach to assess the strength of evidence for competing models. To fit the models, we used the ‘nlme’ package for linear and non‐linear mixed effects models in R (Pinheiro *et al*. 2012; R Development Core Team 2012). Our models included fixed effects (the parameters *a* and/or *b*) and a random factor (‘Site’) to account for average differences in the aggression scores among sites (there were three different sites per resource level; see Pruitt & Goodnight (2014a,b) for details). For each fitted model, we recorded Akaike's information criterion (AIC; Akaike 1973) and, following Burnham & Anderson (2002), calculated the following statistics for the *i*th model in the set of *N* competing models (*i *∈ 1, 2, …, *N*): (1) the AIC difference, Δ_*i*_ = AIC_*i*_ − AIC_min_; (2) the relative likelihood, *l*_*i*_ = exp(−[1/2]Δ_*i*_); and (3) the ‘evidence ratio’ for the best model vs. model *i*, calculated as 1/*l*_*i*_.

## Results

### High‐resource models

**Figure 2:**
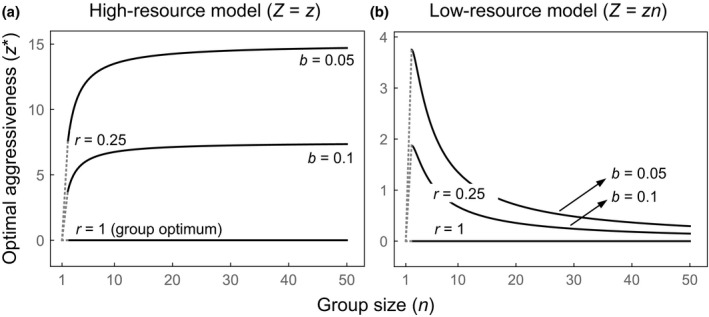
Optimal individual aggressiveness predicted by our high‐resource model (a) and low‐resource model (b), assuming a linear cost of aggression (where *b* measures the rate at which aggression reduces group success). In both models, pure group‐level adaptation (represented by *r *=* *1) favours no aggression; in contrast, multilevel selection (represented by *r *=* *0.25) favours aggression to help individuals compete over local resources. In (a), the individual cost of aggression is lowest in large groups, and so the optimal aggressiveness increases with group size *n*. In (b), the cost of aggression is highest in large groups, and so the optimal aggressiveness decreases with group size (above *n *=* *2). Dotted lines illustrate the change from *n *=* *1 to *n *=* *2.

The high‐resource models predict that under multilevel selection (*r < *1), the optimal aggressiveness z* will increase as group size *n* gets larger (Table 1 and Fig. 2a; Frank 1994). This is because the shared efficiency cost of aggression becomes distributed among a larger number of individuals, so each individual (including the focal individual) incurs a smaller fraction of the costs as group size gets larger. Aggression is inhibited by higher relatedness *r*, and in the extreme case of pure group‐level adaptation (*r *=* *1), a baseline level of aggression is favoured, irrespective of group size (with linear and exponential costs, the baseline is zero; in the ‘humped benefit’ model, the baseline is *b*). Hence, all group‐size‐dependent aggression in this model is due to incorporating selection for individual competitiveness within groups. The effect of group size on z* can be attributed to the increasing relative strength of within‐group selection compared to between‐group selection as group size gets larger.

**Table 1:** Predicted optimal aggressiveness z* for alternative models of spider aggression in high‐ and low‐resource environments, expressed in terms of whole‐group relatedness *R* or others‐only relatedness *r*

Resource level	Model description	z*_R_	z*_r_
High	Linear cost	(1 − *R*)/*b*	([*n* − 1][1 − *r*])/(*bn*)
	Exponential cost	(1 − *R*)/(*bR)*	([*n* − 1][1 − *r*])/(*b*[1 – *r *+ *nr*])
	Humped benefit	*b*/*R*	(*bn*)/(1 + [*n* − 1]*r*)
Low	Linear cost	(1 − *R*)/(*bn*)	([*n* − 1][1 − *r*])/(*bn* ^2^)
	Exponential cost	(1 − *R*)/(*bnR*)	([*n* − 1][1 − *r*])/(*bn*[1 – *r *+ *nr*])

### Low‐resource models

The low‐resource models predict that under multilevel selection (*r < *1), the optimal aggressiveness z* will increase as group size gets smaller (with a maximum at *n *=* *2; Table 1 and Fig. 2b; Godfray & Parker 1992). This is because, although within‐group conflict promotes aggression, the cost for a given level of aggression (group extinction risk) increases with group size, and this selects against aggression in large groups. The extreme case of pure group‐level adaptation (*r *=* *1) favours a low baseline level of aggression, irrespective of group size (with linear and exponential costs, the baseline is zero). Hence, as above, all group‐size‐dependent aggression is due to incorporating selection for individual competitiveness within groups. Here, however, the effect of group size on z* can be attributed to the increasing relative strength of between‐group selection compared to within‐group selection as group size gets larger.

### Model fitting and assessment

**Figure 3:**
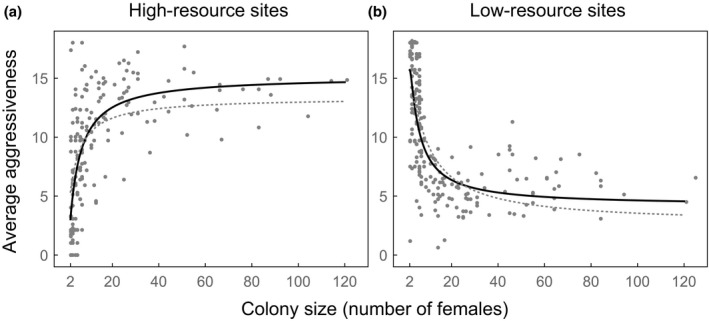
Data on spider aggression in high‐resource sites (a) and low‐resource sites (b) match the predictions from our optimality models (assuming multilevel selection, with *r *=* *0.25). The data shown are the raw average individual aggressiveness scores per colony, with data from three different sites pooled together in each panel. In (a), the solid black line is the best‐fit optimality model (exponential cost, with *b *=* *0.20 [95% CI: 0.19, 0.21]), and the dotted grey line is the next‐best model (humped benefit, with *b *=* *3.3 [3.1, 3.5]). In (b), the solid black line is the best‐fit optimality model (linear cost, with *a *=* *4.2 [3.8, 4.6] and *b *=* *0.016 [0.014, 0.018]), and the dotted grey line is the next‐best model (exponential cost, with *a *=* *2.6 [1.6, 3.6] and *b *=* *0.031 [0.027, 0.035]).

We present results of the model fitting and assessment in Table 2 and Fig. 3. We first compared the models and data for high‐resource sites. We found that the best‐fitting model is the exponential cost model (solid black line, Fig. 3a). This model is thousands of times more likely (given the data) than the next‐best model (humped benefit; dotted grey line, Fig. 3a) and more than a billion times more likely than the linear group adaptation model from Pruitt & Goodnight (2014a) (not shown). Using the models and data from low‐resource sites, we found that the best‐fitting model is the linear cost model (solid black line, Fig. 3b). This model is about sixty thousand times more likely (given the data) than the next‐best model (exponential cost; dotted grey line, Fig. 3b) and more than a billion times more likely than the linear group adaptation model from Pruitt & Goodnight (2014a) (not shown). In sum, our best‐fitting optimality models have exceedingly high empirical support relative to the current model used to explain *A. studiosus* aggression.

**Table 2:** Assessing the strength of evidence for alternative models of optimal spider aggression in high‐ and low‐resource environments

Resource level	Model description	Model notation (using *r *=* *1/4)	AIC_*i*_	Δ_*i*_	Evidence ratio b
High	Exponential cost	(3[*n* − 1])/(*b*[3 + *n*])	1149.1	0	––
	Humped benefit	(4*bn*)/(3 + *n*)	1166.3	17.2	5431
	Linear cost	(3[*n *− 1])/(4*bn*)	1200.8	51.7	> billion
	Linear group adaptation (P&G)^a^	*a *+ *bn*	1236.1	87.0	> billion
Low	Linear cost	*a* + (3[*n *− 1])/(4*bn* ^2^)	1172.6	0	––
	Exponential cost	*a* + (3[*n *− 1])/(*bn*[3 + *n*])	1194.7	22.1	62 944
	Linear group adaptation (P&G)	*a *+ *bn*	1301.3	129	> billion

a P&G denotes Pruitt & Goodnight (2014a).

b The evidence ratio measures how many times less is the empirical support for model *i* compared to the best model in the set of competing models.

## Discussion

Group‐level aggression in the spider *A. studiosus* varies strongly with group size in a manner that depends upon the resources available at a particular site. Our analyses show that these patterns can be explained by within‐group competition, where the costs of this competition differ depending on the resources available to a group. In all of our models, we assume that aggression is a selfish trait that helps an individual compete for and gain resources. However, when resources are plentiful and aggression reduces the shared foraging efficiency of the group, it pays to be most aggressive in large groups (Fig. 2a). In contrast, when resources are scarce and aggression depletes the total amount of food available, it pays to be aggressive only in small groups (Fig. 2b). The striking fit of our predictions to data from natural *A. studiosus* populations suggests that consideration of within‐group competition is crucial for understanding why aggression varies in this species (Fig. 3). In contrast to the group adaptation hypothesis (Pruitt & Goodnight 2014a), our results suggest that aggression is favoured primarily because it is good for the individual, not the group.

Can our models also explain the patterns of colony extinction in experimental populations of *A. sudiosus*? Pruitt & Goodnight (2014a) found that in low‐resource sites, large, aggressive colonies tended to go extinct. This is consistent with our low‐resource models, which assume that colonies with high total aggression (high *zn*) have highest probability of extinction. However, in high‐resource sites, large, docile colonies tended to go extinct, and this appears inconsistent with the assumption of our best‐fitting high‐resource model (‘exponential cost’ model), in which colonies with low aggressiveness (low *z*) do best. We do not yet know the exact form of these cost and benefit functions, and they may be complex – potentially including a group size‐dependent benefit of defending against social parasites (Pruitt & Riechert 2011; Pruitt 2012; Wright *et al*. 2014). However, we found that incorporating group defence in a simple way (our ‘humped benefit’ model) does not alter our main conclusions. Within‐group competition is still central to understanding the effect of group size on the optimal aggressiveness.

Our work suggests several ways to improve the current understanding of *A. studiosus* ecology and evolution. First, related to the points above, we need more data on how group success varies with group‐level aggression traits. Ideally, this would include measures of colony survival and fecundity and would involve the full range of colony sizes observed in nature. Second, greater attention should be paid to the significance of within‐group competition over resources. Although Pruitt & Goodnight (2015) have claimed that aggressive individuals do not have a competitive advantage over docile individuals, our results – in addition to previous evidence (Pruitt *et al*. 2008; Pruitt & Riechert 2009) – suggest that within‐group competition is a key factor that promotes aggression. In contrast, other evidence suggests that docile and aggressive individuals assume complementary roles in the colony (Holbrook *et al*. 2014; Wright *et al*. 2014), so this needs to be reconciled with evidence for within‐group competition.

Furthermore, we need to know more about how spiders apparently express the optimal aggressiveness for their colony size. Perhaps the simplest mechanism would involve a conditional strategy, in which females adjust their aggressiveness via developmental and/or behavioural plasticity, using colony size as a cue (Smallegange & Egas 2015). Alternatively, if aggressiveness is relatively fixed, with distinct aggressive and docile genotypes, then group compositions would have to evolve over time, under a combination of within‐ and between‐group selection. Importantly, for within‐group selection to be effective in this way, colonies would have to be stable and long lived, with multiple generations at a roughly constant colony size. It is not clear that this could account for the close match between the optimal and observed aggressiveness that is suggested by our results.

We hope to have shown here how a pluralistic approach can help to resolve debates in sociobiology (Foster 2009). Our analyses provide strong evidence that both within‐ and between‐group selection are important in natural populations, as expected. Moreover, our models can be viewed from a kin‐ or multilevel selection perspective, and so our conclusions are not linked to any one perspective. While our models show that aggression levels can be influenced by group‐level selection, however, this does not mean that aggression levels are strictly group‐level adaptations (Okasha 2006; Gardner & Grafen 2009; Bourke 2011; Gardner 2015a). More work is needed to understand the link between group selection and group adaptation (Foster 2009). Similarly, the formal equivalence of kin‐ and multilevel selection frameworks does not mean that they always offer equally useful causal representations of the evolutionary process (Okasha 2006, 2015; Marshall 2015). It is possible, then, that future work will reveal details of *A. studiosus* biology which suggest that one framework explains this system better than the other (Krupp 2016).

Finally, we also hope to have shown how debates in sociobiology will benefit from stronger links between ecologically informed models and data. Our analyses go beyond recent criticisms of Pruitt & Goodnight's (2014a) group adaptation hypothesis (Gardner 2015b; Grinsted *et al*. 2015; Smallegange & Egas 2015) by rigorously evaluating the strength of evidence for alternative models of *A. studious* aggression. Our conclusion on the importance of within‐group competition is also strengthened by the fact that we could derive and test starkly different predictions in high‐ and low‐resource environments. This feature of group‐living spiders suggests that they are an excellent system for testing predictions from sociobiology in nature.

## Authorship

JMB performed the mathematical and statistical analyses, JMB and KRF designed the analyses and wrote the paper.
