# Supplementary material for: Ecology and multilevel selection explain aggression in spider colonies
Source: Ecol Lett. 2016 Jun 6;19(8):873–9. doi: 10.1111/ele.12622 (PMC4950442; doi:10.1111/ele.12622)
Supplement: Supplementary file 1 [file ELE-19-873-s001.pdf]

Supporting Information for: "Ecology and multilevel selection explain aggression in spider colonies" (Jay M. Biernaskie and Kevin R. Foster, University of Oxford)

In this supplement, we present an alternative comparison of our optimality models with data on colony size and aggression in the social spider *Anelosimus studiosus* (Pruitt & Goodnight 2014a, b). As explained in the main text, the typical measure of female aggressiveness in this species has a distribution that is continuous and bimodal, leading to the classification of females as either 'aggressive' or 'docile' (Pruitt & Riechert 2009). Consequently, colony-level aggression can be summarized as either the average aggressiveness of individual females in the colony (as analyzed in the main text) or as the proportion of females in the colony that are classified as 'aggressive' (Pruitt & Goodnight 2014a). Here, we consider the latter option and interpret the strategy in our model as a probability of adopting the 'aggressive' phenotype (see also Gardner 2015). All other model details are as described in the main text. As shown in the figure below, this alternative analysis also leads to a strong match between the data and our best-fit optimality models.

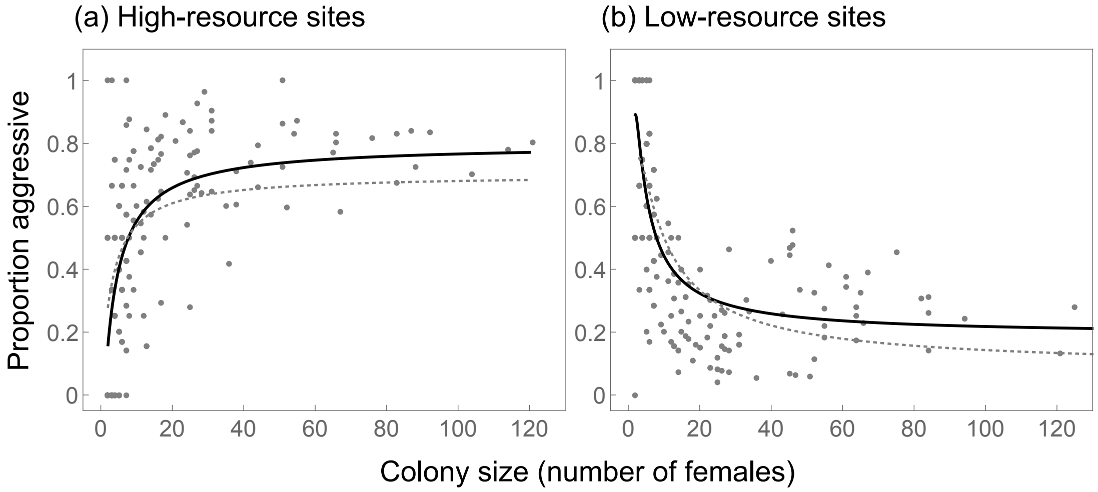

Figure S1. Data on spider aggression in high-resource sites (a) and low-resource sites (b) match the predictions from our optimality models (assuming multilevel selection, with r = 0.25). The data shown are the within-colony proportion of 'aggressive' types, with data from three different sites pooled together in each panel. In (a), the solid black line is the best-fit optimality model (exponential cost, with b = 3.76 [95% CI: 3.53, 3.99]), and the dotted gray line is the next-best model (humped benefit, with b = 0.18 [0.17, 0.19]). In (b), the solid black line is the best-fit optimality model (linear cost, with a = 0.19 [0.10, 0.28] and b = 0.27 [0.22, 0.32]), and the dotted gray line is the next-best model (exponential cost, with a = 0.085 [-0.030, 0.20] and b = 0.50 [0.41, 0.59]).

We note that our model, interpreted in this way, is a simplification of the scenario with two discrete aggression strategies. In particular, it does not account for random variation in the realized number of aggressive and docile individuals that make up a group of size n, which could impact the expected fitness of a focal female. A more explicit model that incorporates this stochastic element could be derived along the lines of Archetti (2009), for example.

1. Archetti, M. (2009). The volunteer's dilemma and the optimal size of a social group. *Journal of Theoretical Biology*, 261, 475–480.
2. Gardner, A. (2015). Group selection versus group adaptation. *Nature*, 524, E3–4.
3. Pruitt, J.N. & Goodnight, C.J. (2014a). Data from: Site-specific group selection drives locally adapted group compositions.
4. Pruitt, J.N. & Goodnight, C.J. (2014b). Site-specific group selection drives locally adapted group compositions. *Nature*, 514, 359–362.
5. Pruitt, J.N. & Riechert, S.E. (2009). Frequency-dependent success of cheaters during foraging bouts might limit their spread within colonies of a socially polymorphic spider. *Evolution*, 63, 2966–2973.
